# Building capacity and capability for science diplomacy: challenges in decolonizing the curriculum for Global Health System Leadership

**DOI:** 10.3389/fpubh.2025.1441351

**Published:** 2025-02-24

**Authors:** Ross Millar, Ayat Abu-Agla, Elsheikh Badr

**Affiliations:** ^1^University of Birmingham, Birmingham, United Kingdom; ^2^Health Services Management Centre (HSMC), University of Birmingham, Birmingham, United Kingdom; ^3^University of Birmingham Dubai, Dubai, United Arab Emirates; ^4^International Academy of Public Health, Amman, Jordan

**Keywords:** science diplomacy, global health, leadership, decolonalization, education, United Arab Emirates, Eastern Mediterranean Region, international branch campuses

## Abstract

Education policy is the front and center of contemporary science diplomacy. This is particularly the case in global health where nation-states are being called upon to equip their health workforce with leadership skills and competencies to improve population health and engage in health system development. In the perspective that follows, we argue that successfully meeting such demands requires not only a high-quality education offering but also one that is aware of and engaged with the coloniality underpinning its underlying assumptions. This perspective makes two significant contributions to further understanding of this area. First, it shares the challenges being faced in developing an education offer for Global Health System Leadership at an International Branch Campus (IBC). Second, it provides an overview of how we are proactively seeking to overcome these challenges. The perspective argues that by engaging in a spirit of dialog and reflexivity, there is scope for decolonizing education science diplomacy.

## Introduction

Much has been made of the potential that science diplomacy perspectives can bring to understanding the knowledge management processes and techniques of nation-states in the international arena (1). Education policy can provide an important fault line for science diplomacy to occur. Higher education institutions from the Global North have proactively built on their reputation, expertise, and financial foundation to promote evidence and education opportunities to be available in the Global South and emerging economy nation-states. International Branch Campuses (IBCs) are a major phenomenon in global higher education to further such endeavors. The University of Birmingham Dubai (2) (UoBD) is indicative of the expansion of IBCs, particularly in the United Arab Emirates (UAE) and the wider Gulf states. Founded in 2018, and now a purpose-built campus accommodating up to 3,000 students, UoBD offers undergraduate and postgraduate education courses in a learning environment ‘fusing the historic Aston Webb buildings in Birmingham with the cultural richness and architectural beauty of Arabic heritage’. The campus also offers research and business engagement opportunities with current priority areas of health and well-being, sustainability, AI, data science, and social innovation (3).

While the potential for knowledge exchange is actively promoted within IBCs such as the UoBD, the evidence base surrounding IBCs shows that there are limitations to achieving such goals. Learning from the experience of global science diplomacy education programs shows that inadequate financial support, insufficient capacity building, and limited access to agenda-setting options place constraints on both host and recipient knowledge exchange (4). The curricula of IBCs are brought into question as they prioritize the vocational demand for subjects such as business and engineering at the expense of courses in the social sciences and liberal arts, or indeed courses that encourage critical inquiry, experiential learning, and leadership development (5).

The cultural and societal impact of IBCs warrants further consideration, with questions raised regarding how IBCs contribute to or disrupt local academic landscapes. For example, whether IBCs reinforce historical power imbalances that privilege Western knowledge production remains an underdeveloped area of investigation. Contributions have also tended to overlook the role of IBCs in the perpetuation of colonial constructs, narratives, and practices (5). Clarke made the important point that Western institutions in non-Western host countries continue to have the most political, economic, and epistemic power, largely owing to the ongoing legacies of colonialism. Overlaid on these dynamics are countries such as the UAE that are engaging in their own science diplomacy as ‘a nation with considerable soft power in the world arena’ (6).

The purpose of this perspective is to present a case study of education that offers an original contribution to scientific diplomacy in the context of global health. Global health policy agendas are facing major challenges in meeting the needs of nation-states. Central to these questions is the development of a workforce with the necessary skills and capabilities to navigate the environmental, economic, political, and cultural dynamics of health system improvement. We argue that responding to such challenges requires not only a high-quality education offering but also one that is aware of and engaged with the coloniality underpinning its underlying assumptions. This perspective makes two significant contributions to further understanding of this area. First, it shares the challenges being faced in developing an education offer for Global Health System Leadership at the UoBD IBCs. Second, it provides an overview of how we are proactively seeking to overcome the challenges being faced. This perspective argues that by engaging in a spirit of dialog and reflexivity, there is scope for decolonizing education science diplomacy in the UAE, the Eastern Mediterranean Region (EMR), and beyond.

## Developing education offered at international branch campuses: the challenge of decolonization

Global health systems are being tasked with restructuring, transforming, and upgrading their health systems and their health workforce to meet the needs of universal healthcare coverage (UHC) and sustainable development goals (SDG). The EMR, in which the UAE is located, is a particular case in point (7). There is an urgent need to respond to the regional public health challenges it is currently facing, with alarming rates of communicable and non-communicable diseases, inequities in access, public health emergencies from armed conflict (8) and climate change, and a COVID-19 pandemic experience that exposed significant workforce shortages, skills, and competencies (9).

In response to these challenges, there are calls to strengthen leadership and management skills and competencies with education and professional development opportunities (10, 11). The launch of the Health Services Management Centre (HSMC) Dubai is supporting these efforts with a mission to improve and transform global health policy and workforce development (12).

A central area of development for HSMC Dubai is an education program that responds to the needs of the UAE, the EMR, and beyond. Our MSc in Global Health System Leadership (13) aims to support health professionals and policymakers in developing their global health leadership capabilities. The course translates into a range of accredited modules that encourage critical thinking and analysis in the areas of global health systems and policy; leadership and management; implementation science; strategic planning; global health economics; and emergency preparedness, response, and resilience.

Critical reflection on the foundational concepts of leadership and management to address health system challenges is actively encouraged with theoretical and comparative analysis explicitly engaging with the EMR and Global South contexts. Case studies and learning vignettes are offered in ways that reflect the specificities of the region with invited external speakers providing regional perspectives. Experiential learning methods and simulation exercises, such as developing a health system strengthening intervention through a health emergency preparedness, response, and resilience plan, are encouraged. The program assessment engages participants in written assignment-based tasks alongside the demonstration of science diplomacy skills such as communication, negotiation, and cross-cultural awareness through group presentations and the development of an e-portfolio.

The course content and ideas have been developed and informed by a range of national, regional, and international consultation and engagement exercises. These deliberations have included the following:

- Stakeholder engagement with the Dubai Health Authority (DHA) to understand local and national needs and priorities. Through these discussions, health systems and policy analysis were identified as key priorities. As a result, efforts led to the launch of a health systems and policy continuous professional development (CPD) course for senior officials and their teams (14). This CPD course informed the development of the health systems and policy module.
- Regional engagement at the third International Conference on Public Health in Africa (CPHIA) with a side event entitled ‘Breaking Barriers: Rebuilding health system leaders for MENA Region and Beyond’. This event brought together global health academics and practitioners to discuss the essential traits of global health system leadership, and the pressing need to review the role and purpose of health systems and the health workforce in meeting universal healthcare coverage (UHC) and the sustainable development goals (SDG) (15).
- Our contribution to the Toward Unity for Health (TUFH) conference ‘Beyond boundaries: Revamping Global Health Systems and Leadership Education and Training for All’ engaged with education specialists to critically analyze our program ideas and the extent to which essential global health functions and capabilities are being demonstrated (16).
- Collaborative engagement with the Health System Global (HSG) Health System Research (HSR) 2024 Pre-Conference in the Eastern Mediterranean Region provided an invaluable opportunity to bring together researchers, educators, and policymakers to critically engage with the challenge of ‘Building Just and Sustainable Health Systems’ (17).

Collaboration to support knowledge production is central to our approach, where we have proactively sought to engage with a diverse set of perspectives and promote a decolonizing perspective on health system leadership.

However, challenges remain, with key elements of decolonization still not fulfilled. Indeed, our IBCs offer is open to perpetuating the colonial education model. Financial costs remain high and are likely to be only accessible to those in high socioeconomic groups. Admission criteria are tightly tied to English language competencies and possible visa restrictions, meaning that it is likely to exclude certain workforce populations that the course is looking to support. The course will be delivered exclusively in English and will encompass academic materials that will be predominantly in English, precluding non-native language instruction and bilingual education. The promotion of diversity and inclusivity of knowledge types is central to our course; however, the content is still likely to draw on predominantly Western-based leadership and management theories to inform the learning environment.

The governance and policy frameworks that surround our work at IBCs may also influence how far we can contextualize our offer. Engaging existing faculty and working with the ‘home’ institution context to instill a different approach can pose practical challenges with limited engagement and capacity to support the IBC offer. Working in the UAE context is requires navigating different norms, rules, and regulations that may challenge academic freedoms in the UK or elsewhere. Responding to the host education context and its requirements also needs to be considered to ensure that our offer complies with educational licencing authorities. Balancing competing interests and accountabilities requires sensitivity and priority setting that may be at odds with the creation of authentically developed learning environments that are tailored to different contexts.

We take the point from Flink (18) that working in contexts with authoritarian states, and stakeholder organizations with a for-profit orientation can lead to contexts where ‘normatively stylized concepts’ such as global health can be adapted to seize public legitimacy or whitewash actions. In the following section, we assess a possible way forward that navigates such dynamics.

## A way forward

While the ethos of our global health system leadership program is decolonizing in terms of its mission and content, our reflection on the journey so far identifies notable challenges and tensions that run deep within our education offer. As a member of a UK IBC we are mindful of the privileged position we are starting from and the knowledge and power that carries within education settings. Clarke (5) has pointed out that transnational education (TNE), such as the introduction of IBCs, has made many positive contributions to cross-cultural knowledge; however, they cannot be disentangled from the complex power relationships between participating countries and their institutions.

That said, we are hopeful that our processes and principles are engaging with decolonization. We are encouraged by the viewpoint that by promoting an ethos of continued dialog and reflexivity (19), an education offer can be a catalyst to raise awareness and empower others to decolonize pedagogy and reflect on the challenges faced in doing so.

An important next step from our perspective is to also ensure that these discussions take place outside of the classroom and engage with the frontline workforce. For example, we are working with the International Academy of Public Health (20) (IAPH) on initiatives to scale up public health workforce capacity across the EMR. This conceptual underpinning has informed the strategy adopted by the IAPH and partners to develop an EMR public health competency framework and professional development program. Working examples of relevance to science diplomacy include a focus on the competencies of diplomacy, dialog, and political skills required to work in the region. An emphasis on public health professionals leading work-based research to inform local policy, and emergency and conflict situation training and development is also encouraged.

We are working with Health Systems Global (HSG) to engage global networks in a discussion about decolonizing knowledge for just health systems (21). The accumulated knowledge is being translated into a repository of examples and learning about capacity strengthening for decolonizing health policy and systems teaching and learning strategies, including pre-professional and continuing professional education (22).

Learning from the experience of the IAPH shows the importance of developing a competency framework for public health education that engages with local public health knowledge and understanding alongside the international evidence base. Our learning from engaging with HSG networks also underlines the importance of partnership working and embedding a systems-thinking approach to underpin these various efforts. We have a challenging road ahead in the expansion of our Global Health System Leadership offer, but through proactive engagement with critical reflection, collaboration, and open dialog, we can take steps toward decolonization at IBCs, both in the UAE and beyond.

## Data Availability

The original contributions presented in the study are included in the article/supplementary material, further inquiries can be directed to the corresponding author.

## References

[1] Doğan EÖ Uygun Z Akçomak I. Can science diplomacy address the global climate change challenge? Env Pol Gov. (2020) 31:31–45. doi: 10.1002/eet.1911, PMID: 39888011

[2] University of Birmingham. (2025). Dubai campus history and heritage. Available online at: https://www.birmingham.ac.uk/dubai/about/history-heritage (Accessed September 2, 2025).

[3] University of Birmingham. (2025). Research that matters. Available online at: https://www.birmingham.ac.uk/dubai/research (Accessed September 2, 2025).

[4] Mauduit J-C Gual Soler M. Building a science diplomacy curriculum. Front Educ. (2020) 5:138. doi: 10.3389/feduc.2020.00138, PMID: 39882452

[5] Clarke L. "To educate and liberate?" Moving from Coloniality to Postcoloniality in the International Branch Campus Model. J Comparat Int Higher Educ. (2021) 13:15–35. doi: 10.32674/jcihe.v13i5.3655

[6] Antwi-Boateng O Alhashmi AA. The emergence of the United Arab Emirates as a global soft power: current strategies and future challenges. Econ Polit Stud. (2021) 10:208–27. doi: 10.1080/20954816.2021.1951481, PMID: 39845729

[7] WHO. (2025). WHO Regional Office for the Eastern Mediterranean. Available online at: https://www.emro.who.int/entity/about-us/index.html (Accessed September 2, 2025).

[8] World Health Organization. Build back fairer: Achieving health equity in the eastern Mediterranean region: Report of the commission on social determinants of health in the eastern Mediterranean region. Cairo: World Health Organization (2021).

[9] Abubakar A Khan W Abou El Naja H Al Ariqi L Bélorgeot VD Hauck SJ. COVID-19 pandemic response in the WHO eastern Mediterranean region. BMJ Glob Health. (2022) 7:e008782. doi: 10.1136/bmjgh-2022-008782 PMC9234429 35750341

[10] Al-Mohaithef M Javed NB Elkhalifa AM Tahash M Chandramohan S Hazazi A. Evaluation of public health education and workforce needs in the Kingdom of Saudi Arabia. J Epidemiol Glob Health. (2020) 10:96–106. doi: 10.2991/jegh.k.191123.001, PMID: 32175716 PMC7310808

[11] Nuwayhid I Krisht G Jabbour S DeJong J Zurayk H. Mapping university-based master of public health programs in the Arab world. Annals Glob Health. (2021) 87:3297. doi: 10.5334/aogh.3297, PMID: 34327117 PMC8300585

[12] University of Birmingham. (2025). Health Services Management Centre. Available online at: https://www.birmingham.ac.uk/dubai/departments/health-management (Accessed September 2, 2025).

[13] University of Birmingham. (2025). Available online at: https://www.birmingham.ac.uk/dubai/study/courses/postgraduate/global-health-system-leadership#:~:text=This%20programme%20will%20equip%20you,Responsible%20Leadership%20and%20Management (Accessed September 2, 2025).

[14] University of Birmingham. (2025). Knowledge and evidence. Available online at: https://www.birmingham.ac.uk/dubai/departments/health-management/knowledge-and-evidence (Accessed September 2, 2025).

[15] CPHIA. (2023). Available online at: https://cphia2023.com/event/health-service-management-centre-dubai-university-of-birmingham-breaking-barriers-rebuilding-health-system-leaders-for-mena-region-and-beyond/ (Accessed September 2, 2025).

[16] University of Sharjah. (2023). The Network: Towards Unity for Health and University of Sharjah. Available online at: https://old.sharjah.ac.ae/en/Media/Conferences/tufh2023/Pages/default.aspx (Accessed September 2, 2025).

[17] University of Birmingham. (2024). HSR 2024 Pre-Conference in the Eastern Mediterranean Region: Building Just and Sustainable Health Systems. Available online at: https://healthsystemsglobal.org/event/hsr-2024-pre-conference-in-the-eastern-mediterranean-region-building-just-and-sustainable-health-systems/

[18] Flink T. Taking the pulse of science diplomacy and developing practices of valuation. Sci Public Policy. (2022) 49:191–200. doi: 10.1093/scipol/scab074 (Accessed September 2, 2025).

[19] Shahjahan RA Estera AL Surla KL Edwards KT. “Decolonising” curriculum and pedagogy: a comparative review across disciplines and global higher education contexts. Rev Educ Res. (2022) 92:73–113. doi: 10.3102/00346543211042423

[20] IAPH. (2025). The International Academy of Public Health. Available online at: https://iaph.org/en (Accessed September 2, 2025).

[21] Health Systems Global (HSG). (2024). Building Climate-Resilient Health Systems: The Focus of the 2024 Global Health Systems Symposium. Available online at: https://healthsystemsglobal.org/news/building-climate-resilient-health-systems-the-focus-of-the-2024-global-health-systems-symposium/#:~:text=The%20sub%2Dtheme%20%E2%80%9CKnowledge%20for,to%20join%20this%20unique%20platform (Accessed September 2, 2025).

[22] HSR. (2024). HSR 2024 Program Brochure.pdf – Google Drive Program and speakers – HSR Global Symposium on Health Systems Research.

